# The accuracy and precision of radiostereometric analysis in monitoring tibial plateau fractures

**DOI:** 10.3109/17453674.2010.487930

**Published:** 2010-07-16

**Authors:** Lucian B Solomon, Aaron W Stevenson, Stuart A Callary, Thomas R Sullivan, Donald W Howie, Mellick J Chehade

**Affiliations:** ^1^Discipline of Orthopaedics and Trauma, University of Adelaide, and Department of Orthopaedics and Trauma, Royal Adelaide Hospital; ^2^Discipline of Public Health, University of Adelaide, AdelaideAustralia

## Abstract

**Background and purpose:**

The application of radiostereometric analysis (RSA) to monitor stability of tibial plateau fractures during healing is both limited and yet to be validated. We therefore evaluated the accuracy and precision of RSA in a tibial plateau fracture model.

**Methods:**

Combinations of 3, 6, and 9 markers in a lateral condyle fracture were evaluated with reference to 6 proximal tibial arrangements. Translation and rotation accuracy was assessed with displacement-controlled stages, while precision was assessed with dynamic double examinations. A comparison of error according to marker number and arrangement was completed with 2-way ANOVA models.

**Results:**

The results were improved using more tantalum markers in each segment. In the fracture fragment, marker scatter in all axes was achieved by a circumferential arrangement (medial, anterior, and lateral) of the tantalum markers above the fixation devices. Markers placed on either side of the tibial tuberosity and in the medial aspect of the fracture split represented the proximal tibial reference segment best. Using 6 markers with this distribution in each segment, the translation accuracy (root mean square error) was less than 37 μm in all axes. The precision (95% confidence interval) was less than ± 16 μm in all axes in vitro. Rotation, tested around the x-axis, had an accuracy of less than 0.123° and a precision of ± 0.024°.

**Interpretation:**

RSA is highly accurate and precise in the assessment of lateral tibial plateau fracture fragment movement. The validation of our center's RSA system provides evidence to support future clinical RSA fracture studies.

## Introduction

Radiostereometric analysis (RSA) has been applied to many areas of both clinical and research orthopedics, including the assessment of fracture segment stability during healing (Kopylov et al. 2001, Mattsson and Larsson 2003, Madanat et al. 2006, Chehade et al. 2009). However, the use of RSA to monitor repair in tibial plateau fractures has been limited to 1 study (Ryd and Toksvig-Larsen 1994).

A preliminary evaluation of any analytical technique involves an assessment of the nature and extent of potential measurement errors (Allen et al. 2004). The accuracy and precision of RSA has been validated previously with mathematical analyses (Yuan and Ryd 2000), test-retest investigations (Ryd et al. 2000), and phantom studies (Valstar et al. 2000 Onsten et al. 2001, Bragdon et al. 2002, Allen et al. 2004, Makinen et al. 2004). To date, in vitro phantom studies have used displacement control via translation or rotation stages. Considerable disparity exists between the 5 methods that have been published regarding displacement-controlled accuracy and precision assessment of RSA in total hip arthroplasty and distal radius fracture models (Onsten et al. 2001, Bragdon et al. 2002, 2004, Madanat et al. 2005, Cai et al. 2008). Variations in micrometer resolution, in methods of image acquisition, in software analysis, and in the presentation of statistical results make direct comparisons between these studies difficult.

The high accuracy and precision of RSA allows small cohort studies to be performed (Valstar et al. 2005). However, in order to obtain an objective view of the performance of RSA systems, validation of the technique is necessary. It has been suggested that individual centers employing the RSA technique should validate their own RSA system in a standardized manner using a phantom model (Valstar et al. 2005). To further facilitate the comparison of reported outcomes, a standardized method of reporting of RSA results has been proposed (McCalden et al. 2005, Valstar et al. 2005). McCalden et al. (2005) suggested that RSA accuracy and precision should be presented as the root mean square error (RMSE) and the 95% confidence interval (95% CI), respectively. However, Valstar et al. (2005) suggested that the accuracy and precision of RSA should be presented as the mean, median, and 95% CI. It has been suggested that RSA reports should quote all of these outcomes for each test of accuracy and precision (Valstar et al. 2005).

Inadequate number, inadequate configuration, and the potential instability of implanted tantalum markers are the most important limiting factors to achieving highly accurate and precise RSA measurements (Kärrholm 1989). By investigating the influence of marker placement on the assessment of accuracy and precision of RSA in tibial plateau fractures, an optimal intraoperative marker arrangement will be defined. This, in turn, will ensure that clinical measurements are achieved with the greatest accuracy. Application of a validated standardized marker arrangement will then allow consistent and reliable analysis of lateral tibial plateau fractures monitored by RSA across different healthcare centers. To our knowledge, there have been no published evaluations of the accuracy and precision of RSA in a tibial plateau fracture model.

In order to validate the accuracy and precision of our center's RSA system, the aims of this study were 3-fold. The first was to investigate the influence of the number of markers and their arrangement on the accuracy and precision of RSA in the context of a lateral tibial plateau fracture internally fixed with a buttress plate and screws in vitro. The second was to establish a guideline for the intraoperative marker positioning in this fracture model. The third aim was to determine the in vivo precision of RSA in lateral tibial plateau fractures.

## Methods

**Figure 1.**
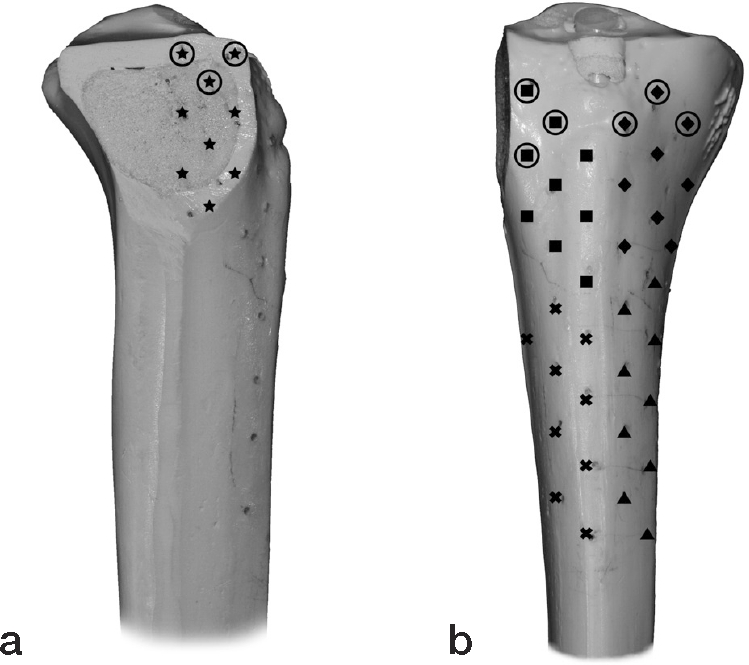
Lateral view (panel a) and anterior view (panel b) of the tibia showing marker placement in the reference segment. ★ segment A, ▪ segment B, ♦ segment C, ▴ segment D, **×** segment E, ○ segment F.

A synbone model of the right tibia was used (Model 1149; Synbone AG, Malans, Switzerland). A split fracture of the lateral tibial plateau was created by osteotomizing the lateral tibial condyle in the sagittal plane. 45 tantalum markers (RSA Biomedical AB, Umeå, Sweden) with a diameter of 1.0 mm were inserted into the proximal tibia using a drill and a spring-loaded piston (RSA Biomedical). The markers were distributed in a matrix of parallel lines 10 mm apart and subdivided into 5 segments: A–E. Segment A was placed on the medial aspect of the fracture split. Segments B–E were placed on the anteromedial and anterolateral aspect of the tibia (Figure 1). Segment B was superolateral, segment C superomedial, segment D inferolateral, and segment E inferomedial on the proximal tibia. All segments contained 9 markers; however, the number of markers per column and row differed slightly to accommodate bone morphology. An additional segment F was also analyzed. This reference segment was a combination of 3 markers from segments A, B, and C. The insertion holes were sealed with a glue to ensure that there would be no movement of the tantalum markers.

**Figure 2.**
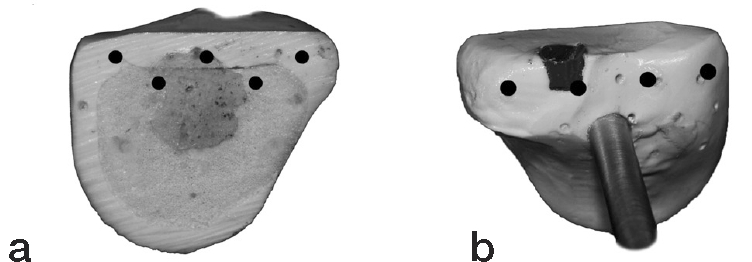
Medial (a) and lateral (b) views of fracture fragment demonstrating marker placement on the fracture fragment. The solid circle represents the fracture segment markers visible after reduction and fixation.

18 tantalum markers of 0.8 mm diameter were inserted into the fractured lateral condyle fragment using the same method as previously described. 2 segments were constructed, each with 9 markers. The first segment (solid circle) occupied the medial, anterior, and lateral aspect of the superior portion of the fracture fragment, while the second segment occupied the inferior portion (Figure 2).

**Figure 3.**
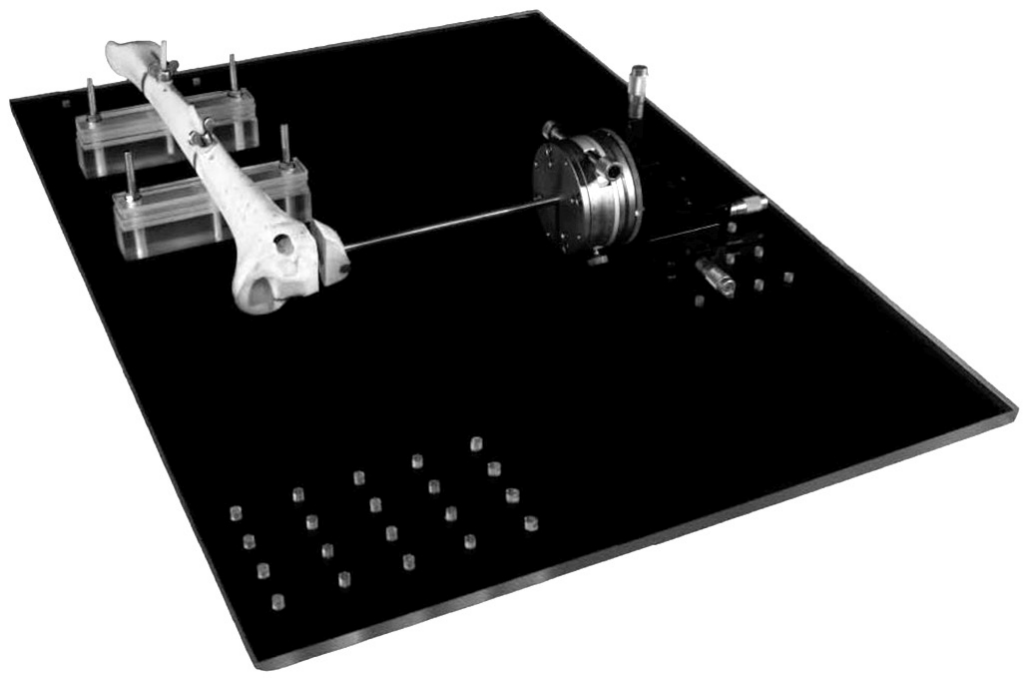
Synbone model of a lateral tibial plateau fracture attached to a translation and rotation stage.

The fracture fragment was rigidly attached to a high-precision x-, y-, z-translation stage (Model M-460A-xyz; Newport, Irvine, CA) by a brass rod connected to a high-precision rotation stage (Model M-UTR-80; Newport). The translation stage was instrumented with 3 Vernier micrometers (Model SM-13; Newport). According to the manufacturer, this translation system is accurate to 1 μm with an angular deviation of less than 150 μrad. The rotation system is accurate to 4 arc seconds with a wobble of ± 60 μrad. Backlash was eliminated by spring-loading the moving assemblies against the tips of the actuators. The tibial shaft was rigidly fixed to a base plate (Figure 3). The fracture fragment was aligned with the tibia, maintaining a 1-mm clearance between the reduced fracture fragment and the tibia.

A uniplanar-type RSA set-up was used with 2 radiographic tubes. A room-mounted unit (Philips Bucky Diagnost) and a mobile radiographic unit (Philips Practix 8000) were positioned with a 30°-angle between the tubes. The calibration cage (Cage 43; RSA Biomedical) contained two 35 × 43 cm high-resolution digital radiographic cassettes. The distance between each focus to film was 1.6 m. All radiographs were exposed at 60 kV and 10 mAs. The image plates were digitized with an AGFA Centricity CR SP1001 processor. The DICOM images were downloaded as Tagged Image Format (TIF) images at 300-DPI resolution. Each radiographic examination was analyzed using the UmRSA version 6.0 software package (RSA Biomedical). Additional internal quality controls were employed during the software analyses. The spatial configuration of the markers within each segment was assessed by the condition number output as part of the UmRSA software. A lower condition number indicates a larger spread of markers representing that segment. Relative marker motion within each segment was assessed by the mean error of rigid body fitting.

To determine the in vitro precision, 6 sequential RSA film pairs were taken. Both the radiograph tubes and the stage were shifted between each examination, simulating the subtle variance in set-up and patient positioning experienced during longitudinal follow-up.

For the accuracy analysis component of the study, the fracture fragment was assessed during both translation and rotation. First, the fragment was translated through each axis, including the x-axis (lateral movement in the coronal plane), the y-axis (distal movement in the coronal plane), and the z-axis (posterior movement in the transverse plane). The fragment was displaced from point zero to 5 mm in 18 increments in each axis. A film pair was exposed at 0 μm, 20 μm, 40 μm, 50 μm, 60 μm, 80 μm, 100 μm, 150 μm, 200 μm, 250 μm, 300 μm, 350 μm, 400 μm, 450 μm, 500 μm, 1 mm, 2 mm, and 5 mm. The fragment was reduced to point zero before commencing translation in each axis. 54 film pairs were obtained. Finally, the fracture fragment was assessed in rotation. Pivoting about the x-axis in the sagittal plane, the fragment was first displaced from point zero to 6° in clockwise direction and then from point zero to 6° in counter-clockwise direction. The fragment was reduced to point zero prior to commencing counter-clockwise rotation. A simultaneous film pair was exposed at 0.5° increments. 26 film pairs were obtained.

**Figure 4.**
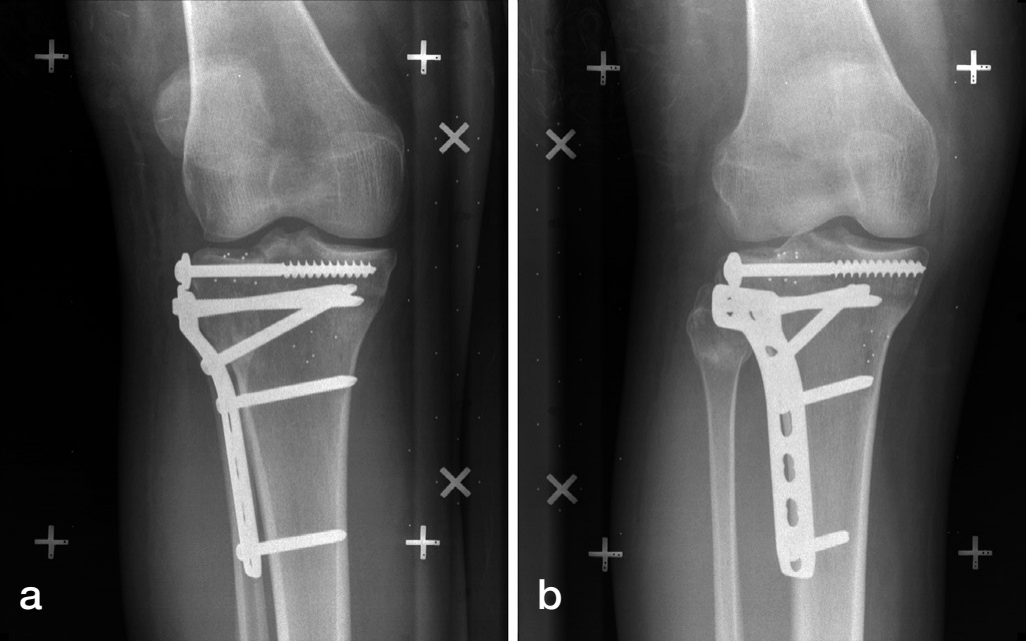
Radiographs of the application of RSA in vivo. Focus 1 (panel a) and focus 2 (panel b).

Following completion of the accuracy and precision assessments, the fracture construct (tibia and fracture fragment) was removed from the stage. The brass rod was cut from the fracture fragment. The fracture was then reduced and fixed with 2 lag screws, a 5-hole Synthes L buttress plate, and 4 cortical screws (Synthes Ltd., Paoli, PA) in a manner concordant with our surgical practice (Figure 4). A final pair of RSA films was taken of the construct after fixation. Markers visible on this image represented those that would allow software analysis in the clinical setting. Only markers visible on the radiographic images following reduction and fixation were included in the accuracy and precision analyses.

The translations and rotation of the fracture fragment were assessed with reference to the 6 proximal tibial segments (A, B, C, D, E, and F). In evaluating optimum fracture fragment marker number and reference segment location, separate calculations of translation accuracy were performed for each possible combination. These combinations included 9 markers in the fracture fragment relative to 9 markers in each tibial reference segment, 6 markers in the fracture fragment relative to 6 markers in each tibial reference segment, and 3 markers in the fracture fragment relative to 3 markers in each tibial reference segment. Precision calculations were performed in a similar manner.

### Statistics

Accuracy was expressed as an RMSE, mean, median, and 95% CI. To compare accuracy according to marker number and position, 2-way ANOVA models were fitted to the data. The difference between the measured value and the true value (the error) was entered as the outcome variable in the models, while marker number and position were entered as the predictor variables. Separate models were performed for the x-, y-, and z-axis translations. Precision analyses were completed for both translations and rotation of the fracture fragment. Precision was expressed as mean, median, and the 95% CI as recommended by the ASTM standard E177-90a. All statistical calculations were performed using SAS version 9.1.

The accuracy and precision results of this phantom model were used to determine a guideline for the number and positioning of markers to represent both the fracture fragment segment and the tibial reference segment in clinical practice. The feasibility of this guideline was tested in 12 patients with 41-B3 (Marsh et al. 2007) and Schatzker II (Schatzker et al. 1979) tibial plateau fractures, in order to determine whether the positioning of these beads was achievable and whether the resultant condition numbers were adequate. To determine the in vivo precision, each patient had two RSA film pairs taken in a supine position within the first postoperative week. The radiographic set-up described above was used for each examination.

## Results

Following reduction and fixation of the fracture fragment in the phantom model, the inferior fragment markers were completely obscured on radiographs by the buttress plate and the screws used to secure it. Thus, subsequent analysis involved only the superior fracture fragment markers (solid circle) in relation to the 6 proximal tibial reference segments (A–F). This split, narrow field of marker distribution represents that which would be achieved clinically during a surgical approach to treat a Schatzker I, II, or III fracture (Schatzker et al. 1979).

The results of different combinations of markers tested for in vitro interfragmentary translation accuracy and precision are presented in Tables 1 and 2. A comparison of error according to the number of markers in the fracture fragment and tibial reference segments was considered for each axis. Regarding the x-axis (lateral movement), there was a difference in error when 3, 6, or 9 markers were used in the fracture fragment (p = 0.003). Post hoc tests indicated that the fracture fragment segment, using either 6 or 9 markers, resulted in lower error compared to using 3 markers (p < 0.001 and p = 0.02 for 6 and 9 markers, respectively). Independently of the fracture fragment segment, there was also a difference in error across the 5 tibial reference segments (p < 0.001). Tibial reference segments C and D had the smallest absolute error. Regarding the y-axis (distal movement), there was a difference in error when 3, 6, or 9 markers were used in the fracture fragment (p < 0.003) and independently across the 5 tibial reference segments (p < 0.001). A smaller absolute error was found using nine markers compared to 3 or 6 markers (p = 0.002 and p = 0.008 for 3 and 6 markers, respectively). 4 tibial reference segments, A, C, E, and F, were associated with a smaller absolute error (p < 0.001). Regarding the z-axis (posterior movement), there was a difference in error when 3, 6, or 9 markers were used in the fracture fragment (p < 0.001) and independently across the 5 tibial reference segments (p < 0.001). Again, using 6 and 9 markers in the fracture fragment and tibial reference segment F was associated with a smaller absolute error.

**Table 1.** Translation accuracy

							Translation accuracy							
Tibial segment			x-axis (lateral)				y-axis (distal)				z-axis (posterior)			
A	B	C	D	E	F	G	D	E	F	G	D	E	F	G
Fracture fragment: 9 markers, condition number 76														
A	9	79	0.017	0.012	± 0.007	0.022	–0.004	–0.005	± 0.006	0.012	0.025	0.027	± 0.007	0.029
B	9	68	–0.036	-0.036	± 0.015	0.047	0.036	0.044	± 0.008	0.040	–0.016	–0.013	± 0.018	0.038
C	9	51	–0.001	–0.003	± 0.008	0.015	–0.005	–0.006	± 0.008	0.016	0.090	0.096	± 0.013	0.094
D	9	107	–0.005	0.000	± 0.015	0.030	0.024	0.031	± 0.010	0.032	–0.044	–0.050	± 0.027	0.070
E	9	71	0.004	0.005	± 0.012	0.025	0.004	0.010	± 0.010	0.020	–0.048	–0.050	± 0.019	0.062
F	9	40	–0.003	–0.002	± 0.009	0.019	0.012	0.006	± 0.018	0.038	–0.003	0.002	± 0.007	0.014
Fracture fragment: 6 markers, condition number 94														
A	6	96	0.014	0.009	± 0.007	0.020	0.010	0.010	± 0.005	0.014	0.023	0.023	± 0.007	0.027
B	6	103	–0.028	–0.032	± 0.017	0.044	0.086	0.090	± 0.011	0.089	–0.009	–0.001	± 0.019	0.040
C	6	66	0.007	0.007	± 0.008	0.017	0.015	0.012	± 0.016	0.035	0.081	0.082	± 0.014	0.085
D	6	130	–0.005	0.014	± 0.019	0.039	0.025	0.029	± 0.010	0.032	–0.011	–0.008	± 0.033	0.068
E	6	121	0.028	0.029	± 0.013	0.039	–0.009	–0.006	± 0.009	0.019	–0.009	–0.011	± 0.017	0.044
F	6	50	–0.007	–0.002	± 0.010	0.021	0.005	–0.001	± 0.018	0.037	–0.005	–0.004	± 0.007	0.015
Fracture fragment: 3 markers, condition number 141														
A	3	148	0.019	0.015	± 0.007	0.024	0.001	–0.001	± 0.006	0.011	0.022	0.024	± 0.007	0.026
B	3	134	–0.107	–0.139	± 0.034	0.127	0.094	0.088	± 0.015	0.099	0.006	0.013	± 0.032	0.066
C	3	133	0.011	0.010	± 0.008	0.018	–0.005	–0.006	± 0.008	0.016	0.060	0.061	± 0.032	0.072
D	3	180	0.002	–0.002	± 0.045	0.091	0.085	0.083	± 0.013	0.088	0.097	0.100	± 0.016	0.102
E	3	169	0.022	0.022	± 0.016	0.039	–0.010	–0.009	± 0.011	0.025	0.019	0.025	± 0.035	0.075
F	3	127	–0.046	–0.050	± 0.012	0.052	–0.021	–0.027	± 0.016	0.038	0.008	0.015	± 0.013	0.027

A Reference

B Number of markers

C Condition number

D Mean

E Median

F 95% confidence interval

G Root mean square error

**Table 2.** Translation precision

						Translation precision					
Tibial segment			x-axis (lateral)			y-axis (distal)			z-axis (posterior)		
A	B	C	D	E	F	D	E	F	D	E	F
Fracture fragment: 9 markers, condition number 76											
A	9	79	0.003	0.003	± 0.012	0.097	0.099	± 0.007	–0.051	–0.049	± 0.024
B	9	68	–0.068	–0.069	± 0.016	0.095	0.095	± 0.009	0.002	0.018	± 0.026
C	9	51	–0.027	–0.033	± 0.012	0.087	0.088	± 0.015	–0.076	–0.071	± 0.043
D	9	107	–0.068	–0.083	± 0.035	0.101	0.104	± 0.011	0.016	0.020	± 0.083
E	9	71	–0.058	–0.061	± 0.018	0.081	0.079	± 0.021	–0.060	–0.054	± 0.031
F	9	40	–0.043	–0.039	± 0.009	0.107	0.106	± 0.006	–0.007	–0.011	± 0.024
Fracture fragment: 6 markers, condition number 94											
A	6	96	0.011	0.011	± 0.011	0.102	0.107	± 0.008	–0.050	–0.045	± 0.022
B	6	103	–0.073	–0.071	± 0.021	0.098	0.091	± 0.018	0.007	0.010	± 0.024
C	6	66	–0.022	–0.023	± 0.011	0.069	0.075	± 0.021	–0.098	–0.094	± 0.041
D	6	130	–0.077	–0.079	± 0.032	0.091	0.094	± 0.012	0.004	0.007	± 0.085
E	6	121	–0.043	–0.040	± 0.023	0.079	0.084	± 0.021	–0.092	–0.072	± 0.074
F	6	50	–0.033	–0.029	± 0.016	0.116	0.116	± 0.004	–0.019	–0.017	± 0.012
Fracture fragment: 3 markers, condition number 141											
A	3	148	0.003	0.000	± 0.012	0.107	0.109	± 0.015	–0.059	–0.056	± 0.027
B	3	134	–0.106	–0.106	± 0.019	0.107	0.100	± 0.021	0.043	0.053	± 0.037
C	3	133	–0.030	–0.025	± 0.014	0.058	0.057	± 0.025	–0.111	–0.128	± 0.052
D	3	180	–0.109	–0.117	± 0.032	0.097	0.096	± 0.021	0.047	0.044	± 0.054
E	3	169	–0.051	–0.060	± 0.033	0.081	0.078	± 0.036	–0.073	–0.071	± 0.069
F	3	127	–0.038	–0.029	± 0.021	0.130	0.131	± 0.005	–0.012	0.001	± 0.028

A–F: See Table 1

Tibial reference segment F performed most consistently, demonstrating a smaller absolute error compared to segments A, B, C, D, and E in both the y- and z-axis. Using 9 markers in the fracture fragment and 9 in the tibial reference segment showed an equal or smaller absolute error compared to either 6 or 3 markers in the x-, y-, and z-axis.

The rotational accuracy and precision for positive and negative rotations around the x-axis for all combinations of markers tested are presented in Tables 3 and 4. A comparison of error according to the number markers in the fracture fragment and the tibial reference segment was considered for rotations. Regarding both negative and positive rotation in the x-axis, there was no statistically significant difference in error when 3, 6, or 9 markers were used in the fracture fragment. Tibial reference segment E had the smallest absolute error for both negative and positive rotation.

**Table 3.** Rotation accuracy

						Rotation accuracy				
Tibial segment			x-axis (negative)				x-axis (positive)			
A	B	C	D	E	F	G	D	E	F	G
Fracture fragment: 9 markers, condition number 76										
A	9	79	–0.11	–0.12	± 0.039	0.125	–0.04	–0.04	± 0.040	0.066
B	9	68	–0.13	–0.14	± 0.040	0.137	–0.06	–0.06	± 0.049	0.086
C	9	51	–0.07	–0.08	± 0.036	0.087	0.00	–0.01	± 0.043	0.058
D	9	107	–0.05	–0.06	± 0.035	0.071	–0.01	–0.03	± 0.041	0.055
E	9	71	–0.05	–0.05	± 0.043	0.076	–0.01	–0.03	± 0.037	0.050
F	9	40	–0.13	–0.13	± 0.037	0.138	–0.07	–0.06	± 0.041	0.092
Fracture fragment: 6 markers, condition number 94										
A	6	96	–0.11	–0.13	± 0.045	0.129	–0.12	–0.11	± 0.034	0.126
B	6	103	–0.13	–0.13	± 0.045	0.145	–0.12	–0.10	± 0.071	0.149
C	6	66	–0.07	–0.08	± 0.045	0.095	0.03	0.02	± 0.037	0.059
D	6	130	–0.01	–0.02	± 0.037	0.050	0.01	0.02	± 0.040	0.055
E	6	121	0.01	–0.00	± 0.041	0.055	–0.02	–0.02	± 0.038	0.052
F	6	50	–0.11	–0.10	± 0.038	0.118	–0.11	–0.10	± 0.037	0.123
Fracture fragment: 3 markers, condition number 141										
A	3	148	–0.10	–0.11	± 0.039	0.109	–0.10	–0.11	± 0.040	0.113
B	3	134	–0.16	–0.14	± 0.061	0.180	–0.09	–0.09	± 0.076	0.139
C	3	133	–0.05	–0.03	± 0.052	0.084	0.04	0.06	± 0.039	0.065
D	3	180	–0.10	–0.11	± 0.037	0.111	0.06	0.06	± 0.046	0.083
E	3	169	–0.04	–0.02	± 0.045	0.072	0.01	–0.02	± 0.057	0.077
F	3	127	–0.14	–0.13	± 0.062	0.164	–0.07	–0.08	± 0.040	0.085

A–G: See Table 1

**Table 4.** Rotation precision

						Rotation precision					
Tibial segment			x-axis			y-axis			z-axis		
A	B	C	D	E	F	D	E	F	D	E	F
Fracture fragment: 9 markers, condition number 76											
A	9	79	–0.16	–0.16	± 0.018	–0.08	–0.08	± 0.047	–0.04	–0.03	± 0.019
B	9	68	–0.12	–0.11	± 0.040	0.10	0.09	± 0.033	–0.04	–0.03	± 0.020
C	9	51	–0.13	–0.11	± 0.032	0.00	0.00	± 0.029	–0.03	–0.03	± 0.029
D	9	107	–0.11	–0.11	± 0.020	0.12	0.13	± 0.085	–0.04	–0.04	± 0.016
E	9	71	–0.11	–0.11	± 0.029	0.02	0.02	± 0.046	–0.02	–0.01	± 0.013
F	9	40	–0.12	–0.13	± 0.028	0.09	0.09	± 0.013	–0.05	–0.06	± 0.017
Fracture fragment: 6 markers, condition number 94											
A	6	98	–0.11	–0.12	± 0.022	–0.09	–0.09	± 0.034	–0.03	–0.02	± 0.024
B	6	103	–0.10	–0.12	± 0.065	0.13	0.14	± 0.050	–0.03	–0.03	± 0.024
C	6	66	–0.11	–0.11	± 0.033	–0.02	–0.02	± 0.036	0.01	0.01	± 0.032
D	6	130	–0.11	–0.11	± 0.025	0.17	0.19	± 0.095	–0.03	–0.02	± 0.023
E	6	121	–0.10	–0.10	± 0.028	0.00	0.00	± 0.064	–0.01	–0.01	± 0.020
F	6	50	–0.09	–0.10	± 0.024	0.07	0.08	± 0.016	–0.06	–0.06	± 0.019
Fracture fragment: 3 markers, condition number 141											
A	3	148	–0.11	–0.12	± 0.031	–0.05	–0.02	± 0.040	–0.05	–0.06	± 0.051
B	3	134	–0.09	–0.11	± 0.084	0.20	0.17	± 0.060	–0.06	–0.07	± 0.063
C	3	133	–0.16	–0.15	± 0.044	–0.01	0.00	± 0.043	–0.02	–0.04	± 0.050
D	3	180	–0.08	–0.08	± 0.021	0.17	0.21	± 0.111	–0.03	–0.02	± 0.051
E	3	169	–0.09	–0.12	± 0.046	–0.00	0.01	± 0.058	–0.03	–0.03	± 0.074
F	3	127	–0.10	–0.09	± 0.033	0.10	0.10	± 0.065	–0.13	-0.13	± 0.045

A–F: See Table 1

All configurations of the fracture fragment and tibial reference segments with 6 and 9 markers had condition numbers less than 150. When configurations with 3 markers were included, the condition number was less than 180. The tolerance mean error of rigid body fitting was 100 μm for both the fracture fragment segment and the tibial reference segments.

From these accuracy and precision results, we propose a guideline that involves insertion of at least 6 markers placed circumferentially (lateral, anterior, and medial) in the fracture fragment, and at least 6 markers placed on either side of the tibial tuberosity and in the medial aspect of the fracture split, which corresponds to segment F in our phantom model. Using this guideline, the corresponding translational accuracy was less than ± 37 μm with a precision of less than ± 16 μm. The accuracy of rotation measurements is less than ± 0.123° with a precision of less than ± 0.024°.

Figure 4 demonstrates the application of this guideline for marker placement in our clinical practice. In this patient, markers were placed on the lateral, anterior, and the medial surface of the fracture fragment. The fracture fragment condition number was 90. Markers were inserted in the tibial tuberosity to represent the tibial reference segment and the resultant condition number of this segment was 120.

The in vivo translation precision for the 12 cases analyzed was ± 98 μm, ± 57 μm, and ± 85 μm in the x-, y-, and z-axis, respectively. The in vivo rotation precision was ± 0.331°, ± 0.260° and ± 0.165° in the x-, y-, and z-axis, respectively.

## Discussion

In our phantom study, the use of 9 markers improved the accuracy of translations in all 3 axes. The accuracy achieved with all 3 combinations of marker numbers used in this study was comparable between themselves and to the results obtained in both distal radial fracture and hip migration studies (Bragdon et al. 2004). Although Madanat et al. (2005) found that 3 markers in each fragment were sufficient, we recommend the placement of 6 markers. This allows for marker exclusion that may occur with post-surgical fracture fragment displacement and in rare cases of intraosseous marker instability.

The accuracy of RSA is dependent on both marker distribution and stability. The distribution of markers can be assessed using the condition number (Soderkvist and Wedin 1993). Segments with condition numbers of less than 150 have been reported to allow reliable RSA results (Valstar et al. 2005). However, condition numbers of greater than 150 have been used when the size of the bone or fragment limits spread of the markers, such as in examinations of the cervical spine, distal radius, and finger joints (Ryd et al. 2000, Madanat et al. 2005). Our method of marker placement in the tibial plateau fracture fragment achieved condition numbers of less than 150 when 6 or 9 markers were used. Integral to this success was the placement of markers on the medial, anterior, and lateral aspect of the fracture fragment. In addition to this circumferential marker placement, positioning above the fixation devices was also necessary to avoid radiological concealment. Subtle variance in coronal height of each marker in the fracture fragment will further improve the analysis performance.

The magnitude of the condition number should always be related to the stability of the markers. The mean error of rigid body fitting is commonly used to assess the marker stability, the upper limit being 0.35 mm (Valstar et al. 2005). As our markers were glued to a synthetic material and were not subject to any load, the mean error of rigid body fitting reported, less than 100 μm, represents the effects of film acquisition, the mathematical algorithm of the software, and the marker model used.

All configurations tested to represent the proximal tibia reference segment were deemed to have acceptable accuracy and precision values. However, our results did find that proximal tibial reference segment F showed the lowest mean error amongst the reference segments. As opposed to segment F, all the other tibial segments are positioned either on the medial or the lateral surfaces of the tibia. Thus, beads placed in segments A, B, C, D, and E will have a more planar (2-dimensional) distribution. Beads were not positioned on the posterior surface of the tibia in the phantom model because of the difficulties the surgeon would encounter in replicating this positioning intraoperatively in a lateral tibial plateau fracture. It is therefore recommended that beads inserted on either side of the tibial tuberosity and in the medial aspect of the fracture split, segment F in our phantom model, be used intraoperatively as the reference segment. Successful clinical application of the marker placement, as illustrated in the case presented in Figure 4, affirms the practical viability of this guideline. An intraoperative image intensifier can confirm correct marker placement in both the fracture fragment and the proximal tibia.

Our center employs a uniplanar type of RSA design. This allows freedom of knee joint examination in flexion, extension, and rotation. The uniplanar design is, however, associated with marker position errors in a direction perpendicular to the radiograph (Yuan and Ryd 2000). This correlates with the anterior-posterior translations (z-axis) in our study of tibial plateau fractures. Cai et al. (2008) also found reduced precision in the z-axis using the uniplanar set-up. The error of translations in this axis was approximated to be 3 times greater than in the x- and y-axis (Yuan and Ryd 2000). The poor performance of the z-axis in the uniplanar design is attributed to “out-of-plane” marker localization. With digital RSA evaluation and appropriate marker spread, the magnitude of this z-axis error may be reduced to that of the x- and y-axis (Borlin et al. 2006). We found that by placing the proximal tibial reference segment (segment F), marker scatter was conferred in the z-axis as both the tuberosity morphology and plane of the fracture split provided relative variation in anterior-posterior marker placement. This spread of markers in conjunction with digital RSA evaluation allowed the z-axis to be analyzed with confidence in the context of the lateral tibial plateau fracture model presented.

A limitation of this study is the use of a phantom tibia with no soft tissue coverage. The lack of soft tissue coverage coupled with inferior synthetic bone density may have altered image quality. Previous reports using phantom models have been completed both with and without soft tissue components (Ryd et al. 2000, Bragdon et al. 2002, Borlin et al. 2006). However, the lack of coverage should have had little influence in the context of the knee, as periarticular soft tissue mass is minimal. Hence, the in vivo precision was investigated in the first 12 patients recruited into a prospective RSA lateral tibial plateau fracture trial. As expected, the in vivo precision was inferior to our “best case scenario” laboratory setting. The in vivo precision of this application of RSA in tibial plateau fractures is comparable to results previously reported for prosthetic wear measurement (Borlin et al. 2006).

RSA allows measurement of inter-fragmentary movement in the order of microns during fracture healing, and may allow measurements of induced movement under load (Chehade et al. 2009, Downing et al. 2008). Accurately defining the stability of tibial plateau fractures following surgical reduction could be important for analysis of the quality of fixation achieved at surgery and the extent of fracture healing, and to guide postoperative management. Our results suggest that RSA may be confidently employed as a highly accurate and precise radiographic measurement tool in the assessment of lateral tibial plateau fractures. The application of our suggested guideline for marker placement should allow consistent and reliable analysis of lateral tibial plateau fractures in multicenter studies. The validation of our center's RSA system provides objective evidence to support the presentation of future clinical RSA results.
